# A cross-sectional study of *Tritrichomonas foetus *infection among healthy cats at shows in Norway

**DOI:** 10.1186/1751-0147-53-39

**Published:** 2011-06-20

**Authors:** Kristoffer Tysnes, Bjørn Gjerde, Ane Nødtvedt, Ellen Skancke

**Affiliations:** 1 Department of Food Hygiene and Infection Biology, Norwegian School of Veterinary Science, P.O. Box 8146 Dep., 0033 Oslo, Norway; 2 Department of Companion Animal Clinical Sciences, Norwegian School of Veterinary Science, P.O. Box 8146 Dep., 0033 Oslo, Norway

## Abstract

**Background:**

In recent years, the protozoan *Tritrichomonas foetus *has been recognised as an important cause of chronic large-bowel diarrhoea in purebred cats in many countries, including Norway. The aim of this cross-sectional study was to determine the proportion of animals with *T. foetus *infection among clinically healthy cats in Norway and to assess different risk factors for *T. foetus *infection, such as age, sex, former history of gastrointestinal symptoms and concurrent infections with *Giardia duodenalis *and *Cryptosporidium *sp.

**Methods:**

The sample population consisted of 52 cats participating in three cat shows in Norway in 2009. Samples were examined for motile *T. foetus *by microscopy, after culturing and for *T. foetus*-DNA by species-specific nested PCR, as well as for *Giardia *cysts and *Cryptosporidium *oocysts by immunofluorescent antibody test (IFAT).

**Results:**

By PCR, *T. foetus*-DNA was demonstrated in the faeces of 11 (21%) of the 52 cats tested. DNA-sequencing of five positive samples yielded 100% identity with previous isolates of *T. foetus *from cats. Only one sample was positive for *T. foetus *by microscopy. By IFAT, four samples were positive for *Giardia *cysts and one for *Cryptosporidium *oocysts, none of which was co-infected with T. foetus. No significant associations were found between the presence of *T. foetus *and the various risk factors examined.

**Conclusions:**

*T. foetus *was found to be a common parasite in clinically healthy cats in Norway.

## Background

During the last decade, the protozoan parasite *Tritrichomonas foetus *has been identified as an important cause of chronic large-bowel diarrhoea in cats, especially among purebred cats in multi-cat households. *T. foetus *was first associated with diarrhoea in cats in the USA [1,2], but has since been reported from diarrhoeic and/or non-diarrhoeic cats in the UK [3,4], Norway [5], Australia [6], Switzerland [7,8], Italy [9], the Netherlands [10], and New Zealand [11]. The many recent reports of this parasite in cats might give the impression of feline trichomoniasis as an emerging disease. However, Stockdale *et al*. [12] suggested that the increasingly frequent diagnosis of *T. foetus *in cats might be due to a rise in the awareness about the parasite among veterinarians and improved diagnostic methods, rather than an actual increase in the incidence.

In Norway, *T. foetus *was originally detected in the uterine contents of a cat with pyometra, as well as in the faeces of three other cats in the same household, one of which had a history of diarrhoea [5]. Following this discovery, *T. foetus *has been diagnosed at the Parasitology laboratory of the Norwegian School of Veterinary Science in faecal samples from several cats in different households, both by microscopy and molecular methods. The majority of these animals have been pedigree cats with chronic diarrhoea [13; Gjerde, unpublished observations). However, the occurrence of *T. foetus *among clinically normal cats in Norway has not previously been examined. Hence, the primary aim of the present study was to use PCR-based methods to determine the proportion of *T. foetus *positive animals among clinically healthy Norwegian cats at cat shows. A secondary aim was to assess the effect of possible risk factors on *T. foetus *occurrence; namely age, weight, former history of gastrointestinal symptoms, geographic origin, number of cats in the household, and concurrent infections with two other enteric parasites; *Giardia duodenalis *and *Cryptosporidium *sp.

## Methods

### Study population and collection of faecal samples

In this cross-sectional study, faecal samples were obtained from cats participating in three different cat shows in Norway (two in the South-eastern part, and one in the South-western part of the country) during the fall of 2009. Before the shows, a letter was sent to 300 attending cat owners, in order to invite them to participate in the study and to give the participating owners instructions on how to collect and submit faecal samples. A questionnaire was distributed along with the letter in order to collect basic information about each participating cat concerning age, gender, breed, number of cats in the household, and previous history of gastrointestinal problems in the individual cat or in the household.

Freshly voided faecal samples were collected by the cat owners immediately before or during the cat shows, and submitted along with a completed questionnaire. After collection, the faecal samples where kept at ambient temperature before being further examined and/or frozen at the Parasitology laboratory at the Norwegian School of Veterinary Science. All participating cats were considered healthy by the owners at the time of sample collection. Moreover, in Norway, cats participating in shows are subjected to a health check by a veterinarian and only cats found healthy are allowed into the show area.

#### Ethics

Owner approval was ensured by a written consent at the end of the questionnaire.

### Examination for *Tritrichomonas foetus*

#### Microscopy and culturing

The 39 samples collected at the two shows in the South-eastern Norway reached the lab within eight hours of collection, and wet mounts in physiological saline were prepared and examined for motile *T. foetus *trophozoites under a microscope at 200 × and 400 × magnification. In addition, approximately 0.1 g from each faecal sample was inoculated into an InPouch™ TF-Feline culturing kit (Biomed Diagnostics, USA) with a small plastic loop. The sealed bags were incubated in an upright position at 37°C, and examined daily under the microscope at 100 × or 200 × magnification for at least 6 days.

The remaining samples, which were collected at a show in South-western Norway (n = 13), were not evaluated by these methods, since it took more than 8 hours for the samples to reach the lab.

#### Molecular examination

All 52 faecal samples were examined for the presence of *T. foetus*-DNA by a PCR-based molecular method. Faecal samples were kept frozen at ÷20°C for no more than 12 weeks before DNA extraction was performed. Genomic DNA was extracted from the faecal samples using QIAmp® DNA Stool Mini Kit (Qiagen GmbH, Germany) according to the modified protocol described by Gookin *et al*. [14].

Genomic DNA was then used in a nested PCR with primer pairs TFR4/TFR3 and TFITS-F/TFITS-R in two separate reactions as previously described [5,14]. Each reaction mixture consisted of 2 μl aliquots of genomic DNA (first reaction) or PCR-product (second reaction), 12.5 μl of HotStarTaq Master Mix (Qiagen GmbH, Germany), 10 pmol of each primer, 4 μg bovine serum albumin, and RNase-free water to make a final volume of 25 μl. Both PCR reactions were initiated with Hot Start at 95°C for 10 minutes; followed by 40 cycles of either 94°C for 30 s, 67°C for 30 s, and 72°C for 60 s (first reaction), or 94°C for 30 s, 57°C for 30 s, and 72°C for 30 s (second reaction); and a final extension at 72°C for 10 minutes. PCR reactions were carried out in an iCycler Thermal Cycler (Bio-Rad, USA). Positive and negative controls (distilled water) were included in each PCR run. PCR products were separated by electrophoresis on 1 per cent agarose gel and visualised under UV light after staining with ethidium bromide to check for appropriately sized products.

Genomic DNA from 5 samples found to be positive for *T. foetus *on gel after the nested PCR described above, was amplified with primers TFR4/TFR3, and products from this reaction were further amplified with the same primer pair. The PCR protocol was as described for the first reaction above. PCR-products from the second round of amplification were purified and sent to Eurofins MWG Operon in Germany for sequencing in both directions in order to verify the presence of *T. foetus *DNA. The identity of these sequences was ascertained by sequence comparisons using the program BlastN of the National Center for Biotechnology Information (http://www.ncbi.nlm.nih.gov/).

### Examination for *Cryptosporidium *oocysts and *Giardia *cysts by IFAT

A small subsample (2-3 μl) from all the 52 faecal samples was examined for *Cryptosporidium *oocysts and *Giardia *cysts by standard immunofluorescent antibody test (IFAT). The subsamples were applied on microscope slides, air-dried, methanol fixed, and stained with FITC-labelled monoclonal antibody (Aqua-glo, Waterborne Inc., New Orleans, USA) and examined by fluorescence microscopy, using the appropriate filters, at 200 × and 400 ×magnification.

### Statistical analysis

Statistical analysis was performed using the software package Stata 11 (Stata Statistical Software, Stata Corporation, College Station, TX, USA). The relationship between weight and PCR status was analysed using a student's t-test. The associations between categorical variables and PCR status were assessed with Fisher's exact test due to the small number of observations within each cell of the two-by-two table. The variables age, number of cats in the household and geographical region were dichotomized and analysed in the same way. The generated categorical variables were "young/adult" with a cut-off at 12 months, "small/large cattery", with a cut-off at four or more cats, and "Eastern-/South-western Norway" using county-level borders. A P-value of ≤0.05 was considered statistically significant and post-hoc power calculation was performed using a two-sided test with alpha = 0.05.

## Results

Of the 300 owners that where given a questionnaire, 47 decided to participate, and submitted faecal samples from a total of 52 cats (17.3%). These cats included 21 females (3 sterilized) and 31 males (12 castrated); 32 cats were adults (> = 12 months), 19 were kittens (< 12 months), and one cat was of unknown age. The mean age was 29.9 months (range 3-144 months). Thirty cats were reported as having had a history of diarrhoea, 21 had no such history of diarrhoea, and no data was available for one cat. The number of cats in each household ranged from 1 to 16 cats. Twenty-four cats were living together with three other cats or less, while 26 cats lived with four cats or more; no data could obtained for two cats. Each litter box was used by 1-8 cats. Participating cats belonged to the following breeds: Abyssinian (3), Bengal (3), Birman (2), British Short Hair (5), Devon Rex (2), Exotic (1), Maine Coon (8), Norwegian Forest Cat (4), Oriental (2), Persian (4), Rag Doll (4), Russian Blue (2), Siberian (3), Somali (2), Sphynx (2), and Turkish Angora (3). Two cats were domestic.

By culturing and microscopy, motile *T. foetus *trophozoites were only detected in one sample (2.6%; 1 of 39 samples). By PCR, 11 of 52 samples (21.2%) from 10 different households (21.3%) tested positive for *T. foetus*-DNA, including the sample that was positive for *T. foetus *using the culturing kit. The 11 positive cats included four females and 7 males. Two of the positive cats originated from households in which a second cat was found to be negative. Nine of the positive cats came from multi-cat households (3-15 cats). Mean age among *T. foetus *positive cats was 20.1 months (range 6-97 months). Cats of the following breeds were positive for *T. foetus*: Bengal (2), British Short Hair (1), Exotic (1), Maine Coon (2), Oriental (1), Somali (1), and Turkish Angora (3).

On agarose gel, no bands were visible after amplification with primer pair TFR4/TFR3 when using genomic DNA extracted from the faecal samples as templates. However, strong bands were obtained when PCR-products from this reaction was used as templates in a second round of amplification with the same primers. Sequencing of the latter products from five samples yielded five identical sequences that proved to be 100% identical to other feline *T. foetus *isolates (GenBank accession nos. AF466749-51, EU569309, GU170216-18 and HM046255), including a sequence from the uterus of a Norwegian cat (GenBank accession no. EF165538). The new sequence from the five isolates has been submitted to GenBank, and has been issued accession no. HM856630 (http://www.ncbi.nlm.nih.gov/nuccore/HM856630). All these feline isolates differ by a single nucleotide substitution (T > C) in the ITS2 region from sequences of *T. foetus *from cattle (GenBank accession nos. AF339736, AY485677-79, AY349189, GU170220, M81842 and U85967). The identity of these sequences was ascertained by sequence comparisons using the program BLAST (Basic Local Alignment Tool) of the National Center for Biotechnology Information (http://blast.ncbi.nlm.nih.gov/)

By IFAT, four cats/samples tested positive for *Giardia duodenalis *cysts and one cat for *Cryptosporidium *sp. oocysts. One of these cats had a concurrent infection with both *Giardia duodenalis *and *T. foetus*.

There was no statistically significant association between weight and PCR status. However, there was a week statistically association between positive PCR status for *T. foetus *and previous history of diarrhoea (p = 0.1). Other parameters without a statistically significant association to a positive PCR status were: age, sex, number of cats in the household, area of origin, and concurrent occurrence of *Giardia duodenalis *and *Cryptosporidium *sp. See Table 1 for the distribution of PCR status by risk factor. The post-hoc power calculation showed that the power to detect a difference between a proportion PCR positive from 0.10 to 0.30 was 20% with alpha (two-sided) set to 0.05. For alpha = 0.1 the power was 30%.

**Table 1:** Relationship between PCR status for *T. foetus *and number of positive individuals with each risk factor, and proportion of PCR-positive cats by category with 95% confidence intervals (CI).

		*T. foetus *PCR			
Variable		Number negative	Number positive	Proportion PCR +	95% CI
Age					
	*> = 12 months*	26	6	0.19	0.047, 0.33
	*< 12 months*	14	5	0.26	0.07, 0.46
Sex					
	*Female*	17	4	0.19	0.01, 0.37
	*Male*	24	7	0.23	0.07, 0.38
Cats in household					
	*< = 3*	20	4	0.17	0.01, 0.32
	*4+*	19	7	0.27	0.10, 0.44
Region where cats lived					
	*East*	23	8	0.26	0.10, 0.42
	*South-west*	18	3	0.14	-0.01, 0.30
*Cryptosporidium*					
	*No*	39	11	0.22	0.10, 0.34
	*Yes*	1	0	No observations	
*Giardia*					
	*No*	38	10	0.21	0.09, 0.33
	*Yes*	3	1	0.25	-0.25, 0.75
History of diarrhoea (n = 51)					
	*No*	19	2	0.10	-0.04, 0.23
	*Yes*	21	9	0.30	0.13, 0.47
Diarrhoea in household (n = 48)					
	*No*	13	1	0.07	-0.07, 0.23
	*Yes*	24	10	0.29	0.14, 0.45

Results based on a cross-sectional study of 52 clinically healthy cats attending cat shows in Norway.

## Discussion

In this study, 11 (21.2%) of 52 faecal samples obtained at three cat shows in Norway were identified as *T. foetus *positive by PCR. This number is lower than the proportion found among cats attending cat shows in USA (36 of 117 cats; 31%) [15] and New Zealand (18 of 22 cats; 82%) [11]. Nevertheless, this is a fairly high proportion considering the fact that all of these Norwegian cats were clinically normal. Some of the previous investigations are not directly comparable with the current study, since they have comprised both diarrhoeic and non-diarrhoeic cats (0% in [6]; 2% in [10]; 10% in [12]), or only cats with chronic diarrhoea (14% in [4]; 24% in [8]), but most of the other frequencies of *T. foetus *positives are lower than what was found in the present study. This might be because the reported surveys comprise a mixture of both purebred and mixed-breed cats, whereas cats attending cat shows are mostly purebred. Pedigree cats appear to be more frequently infected with *T. foetus *than mixed-breed cats [4,12]. However, purebred cats might not be more susceptible to infection with *T. foetus*, but simply more likely to become infected because they often live in multi-cat households and are commonly in contact with cats from other households through breeding and participation at shows [4].

The mean age among infected cats (29.9 months) in this study was higher than that reported by Gookin *et al*. [15] from cats at an international cat show in the USA. Similar to the current study, Gookin *et al*. [15] found no correlation between age and *T. foetus *infection, which supports the notion that cats of all ages might be infected by *T. foetus*. However, young cats seem more likely to have clinical infections with diarrhoea than adult cats [1]. This might be related to lack of previous exposure to *T. foetus *and absence of immunity, because even adult cats might become clinically affected if first exposed at an older age. Thus, Holliday *et al*. [9] reported such infections from a feline rescue colony in Italy where 32% of the cats suffered from *T. foetus *infection.

No significant associations were found between the included risk factors and PCR status for *T. foetus*. However, the power to detect any differences was low (20% at alpha = 0.05) and a larger study group would be required in order to draw any firm conclusions regarding these relationships. Even though 300 questionnaires were handed out, only 47 (15.7%) of the owners decided to let their cat(s) participate in the study. A low response-rate is a potential source of bias and warrants caution when interpreting results. The internal validity (ability to extrapolate results from the study group to the source population of cats at the three cat shows) might be compromised as a result of this potential selection bias. The high number of non-responders may have affected the final result in several ways. Owners which had experienced problems with chronic diarrhoea in their cattery might have been more interested in participating in this study, and this could lead to an over-estimate of the proportion of infected individuals. On the other hand, owners suspecting *T. foetus *in their cattery might have been more reluctant to participate for fear that their reputation could be damaged by a positive result, despite our attempt to inform owners that all results would be kept confidential.

Many owners were unable to submit samples for this study because their cats did not defecate during the show. It has been suggested that *T. foetus *infected cats might be overrepresented when collecting faecal samples at cat shows, because these cats often have an increased urgency to defecate compared with cats not infected by *T. foetus *[16,17].

*T. foetus *is transmitted by the direct faecal-oral route, and might survive for some hours in a moist environment [18] and transmission may occur when cats are sharing litter boxes and when they are grooming each other. In Norway it is common practise for breeders to temporarily exchange cats for reproductive purposes. This exchange may be an important way of spreading the disease. Retrospectively we think that the prevalence of such exchange in each household should have been included in the questionnaire.

*T. foetus *can be a challenging organism to culture due to its susceptibility to low temperatures and dry conditions. In this study, culturing was only performed on samples (n = 39) that had not been subjected to cold temperatures, but only managed to identify one of the 11 samples that tested positive by PCR. Hence culturing does not seem to be a suitable diagnostic method for detection of subclinical *T. foetus *infections, in which the faeces is rather dry. The manufacturer's recommendation for incubation temperatures of the culture system used is between 15 and 37°C, and we chose to incubate at 37°C. In 2003 Gookin et al. performed a study on different protocols using the same culture system and found that incubation at 37°C gave a quicker positive result, but also more bacterial overgrowth that may inhibit *T. foetus *growth. Incubation at lower temperatures made more robust and long lived cultures.

Sequencing of five isolates from the current study yielded five identical sequences consistent with previously published sequences of *T. foetus *in GenBank from cats in Norway, USA and Australia. Moreover, all these new sequences displayed the same single nucleotide polymorphism (T > C) in the ITS2 region, which seems to separate between *T. foetus *of feline and bovine origin, respectively [19]. Experimental infection studies have indicated that there are differences between these strains as regards virulence and infectivity. A feline *T. foetus *isolate was inoculated in the reproductive tract of heifers and caused endometritis, but apparently with less severe clinical signs than lesions caused by a bovine isolates [17]. Similarly, a bovine isolate of *T. foetus *was able to infect the caecum of cats, but seemed to be less infectious for cats than an isolate of feline origin [20].

The current study group was a subset of cats attending three cat shows in Norway during 2009. Since this group was a convenience sample without randomization, the external validity (ability to extrapolate results beyond the source population consisting of cats at these three shows) of the study is low. Therefore, no claims about population parameters such as prevalence can be made based on the current study. However, the results are an important contribution to the body of evidence regarding *T. foetus *occurrence in Norway because this is the first time a large number of healthy cats have been examined for this parasite in this country. The results indicate that *T. foetus *is indeed present even among clinically normal Norwegian purebred cats, and should therefore also be considered as a possible etiologic agent in cats with un-resolving large bowel diarrhoea. In order for veterinarians to address important questions, such as the population prevalence or potential for *T. foetus *transmission outside catteries, further epidemiological investigations are needed.

## Conclusions

*T. foetus *is a rather common parasite in clinically healthy purebred cats in Norway, and should therefore be considered as a possible etiologic agent in such cats with a history of chronic large-bowel diarrhoea.

## Competing interests

The authors declare that they have no competing interests.

## Authors' contributions

All authors participated in the planning of the study, contributed to the writing of the paper, and read and approved the final manuscript. KT organised and conducted the faecal sampling, did the major portion of the laboratory examinations, and had the main responsibility for drafting the manuscript. BG assisted with the laboratory examinations and was responsible for DNA-sequencing of selected samples. AN did the statistical analysis. ES supervised the entire study and helped obtain funding.
